# Role of Radiation Therapy in Adult Extraskeletal Ewing's Sarcoma Patients Treated with Chemotherapy and Surgery

**DOI:** 10.1155/2019/5413527

**Published:** 2019-04-24

**Authors:** Augustine M. Saiz, Alicia A. Gingrich, Robert J. Canter, Amanda R. Kirane, Arta M. Monjazeb, R. Lor Randall, Steven W. Thorpe

**Affiliations:** ^1^Sarcoma Services, MSK Section, Department of Orthopaedic Surgery, UC Davis Medical Center, Sacramento, CA 95817, USA; ^2^Division of Surgical Oncology, Department of Surgery, UC Davis Medical Center, Sacramento, CA 95817, USA; ^3^Department of Radiation Oncology, UC Davis Medical Center, Sacramento, CA 95817, USA

## Abstract

Radiation therapy (RT) is advocated in the multimodal treatment of high-grade soft tissue sarcoma (STS), but its role may be less clear in chemotherapy-sensitive STS such as extraskeletal Ewing sarcoma (EES). The purpose of this study was to determine the role of RT on overall survival (OS) in localized EES adult patients treated with chemotherapy and surgery. Adult patients diagnosed with EES and reported to the National Cancer Database from 2004 to 2014 were evaluated. All patients were treated with surgical resection. Patient demographics, tumor characteristics, treatments received, resection margins, and survival were examined for the 232 patients identified. Using multivariate analysis and Cox proportional hazard analysis, predictors of OS were determined. In the overall cohort, 40 percent of patients received RT and 78 percent received chemotherapy, with 31 percent receiving both. The addition of RT to the patients receiving surgery + chemotherapy did not improve OS (*p* < 0.05). Twenty-four percent of patients who achieved R0 resection after surgery still received RT without any improvement in OS. Patients treated at community cancer centers were more likely to receive additional RT compared with Comprehensive Cancer Centers (*p* < 0.05). In adult EES patients with localized disease treated with chemotherapy and surgery, the addition of RT does not improve overall survival.

## 1. Introduction

Ewing sarcoma consists of a group of tumors characterized by morphologically similar round-cell neoplasms derived from undifferentiated mesenchymal cells with the potential for neuroectodermal differentiation [1–4]. Extraskeletal Ewing sarcoma (EES) is a rare soft tissue sarcoma (STS), with an incidence of 1 in 5–10 million and represents a small portion of the 12,000 annual STS roughly [5–8]. Due to the rarity of EES, few large population-based studies exist that address treatment strategies and associated clinical outcomes, especially in adults [9–11]. The largest studies to investigate EES compared outcomes relative to skeletal Ewing sarcoma without particular analysis of treatment modalities specific to EES [7, 12–19].

Historically, patients with EES were treated with a rhabdomyosarcoma protocol but current evidence suggests that these patients benefit from skeletal Ewing sarcoma protocols instead [20, 21]. The National Comprehensive Cancer Network (NCCN) does not differentiate the treatment of EES from skeletal Ewing sarcoma [22]. Standard of care guidelines for treatment of osseous and extraosseous Ewing sarcoma include multiagent chemotherapy prior to local control treatment for nonmetastatic disease [23]. Accepted local control treatment can consist of (1) R0 resection, (2) R1 resection + radiation therapy (RT), or (3) RT and concurrent chemotherapy [23]. Typically, local treatment with surgery alone is preferred if this can be performed with acceptable morbidity. Ewing sarcoma is more common in young patients where the risk of RT-associated secondary malignancies is higher [24]. In addition, some data may suggest that surgery is favorable compared to RT for Ewing sarcoma [25, 26].

As the multimodal treatment of STS has evolved, limb salvage has become feasible in approximately 95% of cases with RT playing a key role in these multimodal treatment protocols [27, 28]. Recent literature demonstrates that about half of all patients with EES receive RT [29]. However, radiation-associated complications such as joint contracture, muscle atrophy, pathologic fractures, and secondary malignancies occurring in 10 percent to up to 63 percent of patients receive RT for sarcomas [2]. The role of RT in adult STS has recently been reviewed, with a large-scale NCDB analysis demonstrating improvements in R0 resection and overall survival (OS) in STS [30]. The analysis did not differentiate between histologic types of STS. This is important as EES is an STS with an above average favorable response to chemotherapy [8]. The histologic specific impact of RT on survival, especially in chemotherapy-sensitive diseases such as EES, remains unclear.

The purpose of this study was to examine the role of RT on overall survival in adult EES patients treated with chemotherapy + surgery. Using data from the National Cancer Database (NCDB), we aimed to determine predictors for the use of RT and if the addition of RT is associated with increased OS for patients with EES treated with chemotherapy + local control surgery. We hypothesized that the addition of RT does not improve OS in adult EES patients treated with chemotherapy + local control surgery.

## 2. Materials and Methods

Using the NCDB, patients with histological diagnosed EES were identified between the years of 2004 and 2014. EES in the NCDB was classified according to the International Classification of Childhood Cancer and/or the International Classification of Disease for Oncology, third revision. A limitation of this database though is that the presence of EWSR1 rearrangement is not documented. Patients less than 18 years of age and patients with unknown skeletal versus extraskeletal status, surgical margin status, tumor size, vital status, chemotherapy status, and/or radiotherapy status were excluded. Patients with metastases were excluded as well as patients with a histologic grade less than 3 as EES represents a high-grade disease; this represents either an incorrect diagnosis or grading. Overall, 232 patients were included in the final analysis. All patients underwent surgical resection for local control. Variables were based on clinical significance and included age, sex, race, year of diagnosis, facility type, Charlson–Deyo score, grade, tumor size, surgical margins, receipt of chemotherapy, and receipt of radiotherapy to the tumor site. Summary statistics were reported as means with standard deviation.

Standard multivariate analysis was performed to determine the predictors of patients receiving RT. Multivariate logistic regression was performed to evaluate RT as a predictor of R0 resection. Cox proportional hazard models were used to assess the effect of the different variables on OS while controlling for known prognostic factors. OS from time of diagnosis was estimated using the Kaplan–Meier method and expressed as Kaplan–Meier estimates with 95 percent confidence intervals. OS was measured from time of diagnosis to time of last contact or death, in months. Disease-specific survival is not captured in the NCDB dataset. All statistical analyses were performed using Stata version 14 (StataCorp LP, College Station, TX). Significance was set at *p* < 0.05 using a two-tailed *t*-test. All patient information was deidentified and, therefore, exempted from the University of California, Davis, Institutional Review Board approval.

## 3. Results

The patient demographics and clinical demographics of the 232 patients with EES are depicted in Table 1. Overall, 13 percent of patients (31/232) received only surgery, 9 percent (20/232) received surgery + RT, 47 percent (108/232) received surgery + chemotherapy, and 31 percent (73/232) received surgery + chemotherapy + RT. In total, 40 percent of all patients (93/232) received RT and 78 percent of all patients (181/232) received chemotherapy. There were no significant differences in patient demographics between patients that received RT and those that did not.

Multivariate regression analysis for predictors of receiving RT revealed no specific variables that were associated with increased probability of receiving RT other than R1 resection and being treated at the Community Cancer Program (*p* < 0.05). Of patients who underwent an R0 resection, 24 percent still received postoperative RT. Community Cancer Programs were more likely to treat adult EES patients with surgery + chemo + RT compared to Comprehensive Community Cancer Programs, Academic/Research Programs, or Integrated Network Cancer Programs (*p* < 0.008).

**Figure 1:**
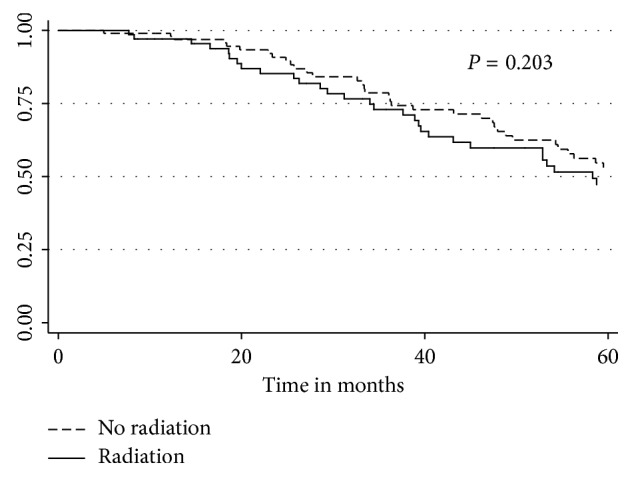
Kaplan–Meier overall survival curves for patients treated with surgery plus chemotherapy with and without radiotherapy.

**Figure 2:**
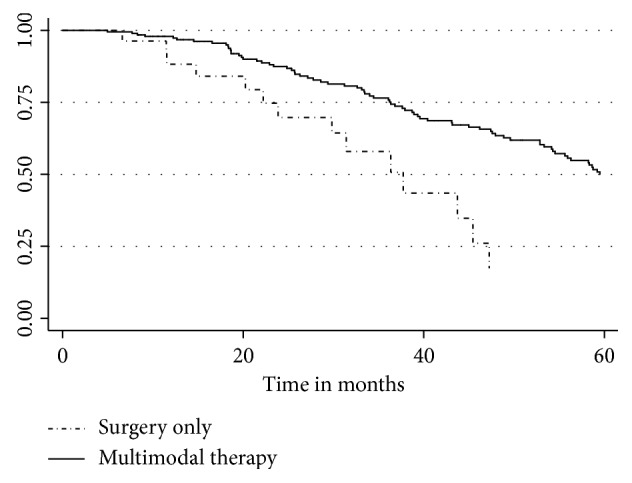
Kaplan–Meier overall survival for patients treated with surgery with and without chemotherapy regardless of radiotherapy treatment.

The Cox proportional hazard analysis for survival demonstrated that a tumor size greater than 10 cm was associated with worse OS (HR 1.93, 95% CI 1.14–3.28, *p*=0.014). R0 resection was not associated with improved OS. In the overall cohort, chemotherapy was associated with improved OS (HR 0.575, 95% CI 0.338–0.978, *p* < 0.041). In the overall cohort, RT was associated with improved OS, but in subset analysis of the 181 patients who received chemotherapy, the addition of radiotherapy was no longer associated with improved survival (Figure 1). Patients who received only surgery had significantly decreased 5-year OS compared to patients who received surgery + chemotherapy ± RT (*p* < 0.032) (Figure 2). In patients who received an R1 resection, radiation therapy was not associated with improved survival (median survival 25.8 months vs. 26.3 months, *p*=0.52).

## 4. Discussion

EES represents a rare high-grade STS, and therefore, limited literature exists examining treatment strategies. Our study represents the first large population-based study, investigating the role of RT in treatment of EES and overall survival in adult patients with localized disease receiving chemotherapy + surgery. A recent large population-based study compared pediatric to adult patients with Ewing sarcoma with subset analysis of extraskeletal locations using the Surveillance, Epidemiology, and End Results (SEER) database and determined that RT was not associated with improved OS in pediatric or adult patients undergoing surgery [31]. There was no analysis of RT in regard to local control. However, chemotherapy was not able to be analyzed as these data are not present in the SEER database [31]. In comparison, the NCDB allowed us to evaluate the role of RT in patients receiving chemotherapy + surgery for local control. This study aimed to specifically examine those patients who received chemotherapy + surgery and determine if RT added extra benefit for OS.

The NCCN standard of care guidelines for treatment of localized EES includes multiagent chemotherapy for at least 12 weeks prior to local therapy with accepted local therapy consisting of (1) R0 resection, (2) R1 resection + RT, or (3) RT and concurrent chemotherapy [23]. Surprisingly, 22 percent of patients did not receive chemotherapy at all; the reason for this deviation from NCCN guideliens is unclear, and further investigation is warranted as chemotherapy is critical for treatment and OS. RT alone is an acceptable treatment for local control as well, but some evidence has suggested that surgery improved outcomes [32]. Ahmed et al. showed that patients who underwent surgical resection, whether wide or less than wide, had improved survival compared to patients who did not have surgery [33]. In accordance with the prior literature, our study did not find an association between R0 surgical resection and overall survival [29, 34–37]. The prior literature noted that 42% of patients with EES receive RT, which correlated with our study where 40% of patients received RT [18]. However, almost 25% of patients with R0 resection received postoperative RT without indication and despite NCCN guidelines. This may be due to some institutions following generalized high-grade STS local therapy algorithms for extraskeletal bone tumors. There is ongoing debate for these tumor types regarding whether skeletal and extraskeletal Ewing should be treated exactly the same. The addition of RT in R0 patients may be related to the finding that Community Cancer Programs were more likely to treat adult EES patients with surgery + chemo + RT than compared to Comprehensive Community Cancer Programs, Academic/Research Programs, or Integrated Network Cancer Programs. Further research investigating the cause of the increased prevalence of RT in addition to surgery and chemotherapy is warranted amongst Community Cancer Programs. Additionally, given that postoperative RT for R0 resection does not improve OS, further examination of why nearly 25% of R0 patients still receive RT in EES is needed. The addition of RT in this subset of patients may represent overtreatment, resulting in risk of increased morbidity. However, other institutions and/or physicians may argue that EES should be treated more as an STS than an osseous tumor in a soft tissue environment, and in that case, RT may be a crucial local therapy agent regardless of outcomes on OS.

The 5‐year OS for all patients in the study was 28.4% but improved to 51% for patients treated with surgery + chemotherapy ± RT. This is consistent with the recent literature demonstrating 5-year OS ranges from approximately 50 to 70 percent [42–45]. Chemotherapy administration was associated with improved OS regardless of resection margin or tumor size. However, the addition of RT to surgery + chemotherapy did not affect OS. Hence, the role of RT with regard to survival outcomes in chemotherapy sensitive, high-grade STS treated with chemotherapy, and local control surgery remains questionable. In the Intergroup Rhabdomyosarcoma Study, of the 130 children with EES, those treated with surgery + chemotherapy + radiotherapy did not have improved OS compared to those treated with surgery + chemotherapy [38]. Our study demonstrates that the vast majority of patients with EES receive chemotherapy + local control surgery, and subsequently, the addition of RT did not improve OS in these patients.

There are important limitations of our study to recognize. First, the NCDB does not contain local control data or disease-specific survival. Secondly, the role of preoperative RT in predicting R0 resection could not be determined due to the low number of patients who received preoperative RT. Specific chemotherapy regimens are not detailed in the NCDB, and different regimens may affect OS [39]. Furthermore, translocation status as a confirmatory diagnostic tool in the Ewing sarcoma family of tumor diagnosis is not available in the NCDB [40]. Finally, a limitation inherent to the NCDB as with other large databases is that the information is self-reported by cancer treatment facilities and may not be reflective of the treatment of EES everywhere.

The strength of our study is the relatively large number of patients with EES in the NCDB. Large studies do exist that examine the differences in patient characteristics and outcomes in patients with EES compared to patients with skeletal Ewing sarcoma, but this study represents the largest study to investigate specifically the role of RT in OS in the EES patient treated with surgery + chemotherapy. Furthermore, our study represents a more modern EES treatment era as our study limits retrospective review of data to 2004 compared to prior studies using the SEER data from as far back as 1973.

## 5. Conclusions

Our study determined that, for adult EES patients with localized disease receiving chemotherapy and surgical resection, the addition of RT did not improve survival although we were unable to assess the effect of RT on local control. Although chemotherapy has been demonstrated to improve OS and is a mainstay of standard of care for EES, 22 percent of patients did not receive chemotherapy at all. Furthermore, the type of treatment facility was an important predictor for the use of RT with community-level programs more likely to use RT. Over 40 percent of patients with EES receive RT, including about 25 percent of patients with negative margins post-op, but without significant improvement in their overall survival.

## Figures and Tables

**Table 1:** Patient demographics and tumor characteristics.

	No. of RTs (144)		Pre-op RT (14)		Post-op RT (79)		*p* value
Age	40.51	±16.85	46.71	±15.36	38.25	±15.46	ns
	N	Percent	N	Percent	N	Percent	
Sex							
Male	72	50.00	9	64.29	43	54.43	ns
Female	72	50.00	5	35.71	36	45.57	ns
Race							
White	130	90.28	12	85.71	71	89.87	ns
Black	8	5.56	0	0.00	3	3.80	ns
American Indian, Aleutian, or Eskimo	2	1.39	0	0.00	0	0.00	ns
Asian	1	0.69	0	0.00	3	3.80	ns
Pacific islander	2	1.39	1	7.14	0	0.00	ns
Others	1	0.69	0	0.00	2	2.53	ns
Grade							
Grade 1	3	2.08	0	0.00	2	2.53	ns
Grade 2	3	2.08	0	0.00	1	1.27	ns
Grade 3	83	57.64	8	57.14	34	43.04	ns
Grade 4	55	38.19	6	42.86	42	53.16	ns
Tumor size (categorical)							
<5 cm	48	33.26	1	7.14	31	39.30	ns
5–10 cm	62	43.02	4	28.57	34	43.08	ns
>10–15 cm	22	15.26	7	50.00	9	11.42	ns
>15 cm	12	8.29	2	14.28	5	6.34	ns
Charlson–Deyo Score							
0	122	84.72	14	100.00	71	89.87	ns
1	18	12.50	0	0.00	7	8.86	ns
>1	4	2.78	0	0.00	1	1.27	ns
Margins							
R0	122	84.72	12	85.71	58	73.42	ns
R1	18	12.50	2	14.29	13	16.46	ns
R2	4	2.78	0	0.00	8	10.13	ns
Chemo							
Not given	31	21.53	5	35.71	15	18.99	ns
Given	108	75.00	9	64.29	64	81.01	ns
Unknown	5	3.47	0	0.00	0	0.00	ns
Facility type							
Community cancer program	2	1.39	0	0.00	3	3.80	ns
Comprehensive community cancer program	17	11.81	2	14.29	10	12.66	ns
Academic/research program	43	29.86	3	21.43	21	26.58	ns
Integrated network cancer program	2	1.39	4	28.57	3	3.80	ns
Unknown	80	55.56	5	35.71	42	53.16	ns

## Data Availability

The datasets generated during and/or analyzed during the current study are not publicly available due to the IRB protocol and restrictions but are available from the corresponding author upon reasonable request.
